# Adjuvant treatment with adipose-derived mesenchymal stem cells (ADSC) reduces severe refractory hemorrhagic cystitis after RIC-PBSCT: A case report

**DOI:** 10.1097/MD.0000000000026316

**Published:** 2021-07-02

**Authors:** Ruixue Yang, Gang Chen, Maria Muhashi, Gulibadanmu Aizezi, Ming Jiang, Hailong Yuan

**Affiliations:** Hematology Center, the First Affiliated Hospital of Xinjiang Medical University, Xinjiang Institute of Hematology, Urumqi, China.

**Keywords:** adipose-derived mesenchymal stem cells, case report, haploid hematopoietic stem cell transplantation, hemorrhagic cystitis, reduced intensity conditioning

## Abstract

**Introduction:**

Severe hemorrhagic cystitis (HC) is still a common complication after allogeneic hematopoietic stem cell transplantation, which affects the quality of life of patients, and may even cause kidney failure. This study reports the clinical effect of adjuvant treatment of adipose-derived mesenchymal stem cells (ADSCs) on severe refractory HC after of reduced intensity conditioning haplotype high-dose peripheral blood hematopoietic stem cell transplantation (RIC-PBSCT) in one case.

**Patient concerns:**

A 53-year-old female patient with acute myeloid leukemia (FLT3-ITD) at high risk received RIC-PBSCT. The patient was relieved with complete donor chimerism of 99.01%, and normal hemogram. However, the patient developed frequent urination, urgency, and dysuria with gross hematuria with blood clots and difficult urinating, especially at night and early in the morning. There were obvious hyperemia and bleeding points in the mucosa of the posterior wall of the bladder.

**Diagnosis:**

The patient was diagnosed as delayed HC of degree IV.

**Interventions and Outcomes:**

The patient was treated with antiviral drugs, urine alkalization, and diuretic drugs for more than 1 month, but no significant effect was obtained. Thus, the patient was then given ADSCs (1 × 10^6^ kg per kg of body weight, infused once a week for a total of 3 infusions). Symptoms of frequent urination, urgency, and dysuria that happened during the first infusion were improved, and blood clots in the urine were also reduced. After the third infusion, HC symptoms disappeared, the red blood cells were normal, and there was no fever, chills, low infusion blood pressure, or rash. The patient's HC was cured. During follow-up, HC recurrence was not observed.

**Conclusion:**

ADSCs adjuvant treatment of relapsed and refractory severe HC is safe and reliable with good clinical efficacy. It shows certain clinical application value, which however requires more clinical cases to further verify this.

## 1 Introduction

Hemorrhagic cystitis (HC) is one of the common complications after allogeneic hematopoietic stem cell transplantation, with the incidence of 3% to 35%.^[1]^ Hematuria (microscopic hematuria and gross hematuria), frequent urination, urgency, and dysuria are the main manifestations. HC is classified into microscopic hematuria (Degree I); gross hematuria (Degree II); gross hematuria with blood clots (Degree III); gross hematuria and blood clots with urethral obstruction (Degree IV).^[2]^ Degree I to III HC is mild, while degree III to IV HC is severe. HC is considered to be mainly related to viral infections. The commonly used treatment methods are antiviral, fluid replacement, alkaline urine, and diuretic treatments, but some patients show poor treatment effects, which may lead to renal insufficiency.^[3–5]^ It is reported that mesenchymal stem cells (MSCs) may be used as adjuvant therapy for severe HC.^[6]^

In this study, a patient with high-risk acute myeloid leukemia received a reduced intensity conditioning hematopoietic stem cell transplantation. The patient developed degree IV HC at 2 months after transplantation. After conservative medical treatment and interventional treatment, HC was not improved. However, a better therapeutic effect was obtained after treatment with adipose-derived MSCs (ADSCs).

## 2 Case presentation

### 2.1 Ethical approval

This study was approved by the Medical Ethics Committee of the First Affiliated Hospital of Xinjiang Medical University. The patient's consent has been obtained in advance for the collection and publication of the relevant data of the paper.

The patient (female, 53 years old, Han nationality) was diagnosed with acute myeloid leukemia (FLT3-ITD) at high risk. In April 2018, the symptoms were not relieved after receiving IDA+Ara-C chemotherapy, but was relieved after being given G-CSF (granulocyte-stimulating factor)+Acla+Ara-C (CAG) chemotherapy. Later, CAG chemotherapy was given again, and a reexamination of the bone marrow showed remission of the bone marrow. HLA typing was performed. In August 2018, the patient received a reduced intensity conditioning HLA haploid-combined peripheral blood hematopoietic stem cell transplantation (RIC-PBSCT). The donor was the patient's son, and the haploid was matched by 5/10. RIC-PBSCT was performed using a reduced intensity conditioning haploid high-dose non-in vitro T-cell-derived PBSCT protocol, which was proposed in 2013 by the Hematology Center. The reduced intensity conditioning protocol was FAB (fludarabine injection, 30 mg/m^2^, -9 to -5 days; cytarabine injection, 2 g/m^2^, -9 to -5 days; Malilan injection, 3.2 mg/kg/day, -4 to -3 days; rabbit anti-human thymocyte immunoglobulin, 2.5 mg/kg, -4 to -1 days). Graft-versus-host response (GVHD) prevention, infection prevention, and supportive care were routinely performed.^[7]^ G-CSF was used to mobilize PBSC of donors for 2 days. The total number of obtained stem cell MNCs was 21.65 × 10^8^/kg, and the total number of CD34+ was 12.84 × 10^6^/kg. At 15 days after transplantation, the donor and recipient gene chimerism status was at complete donor chimerism status with a chimerism rate of 99.28%. The patient's HLA type changed to donor type after 30 days. The rate of complete donor chimerism was 99.01%, and the hemogram returned to normal.

**Figure 1:**
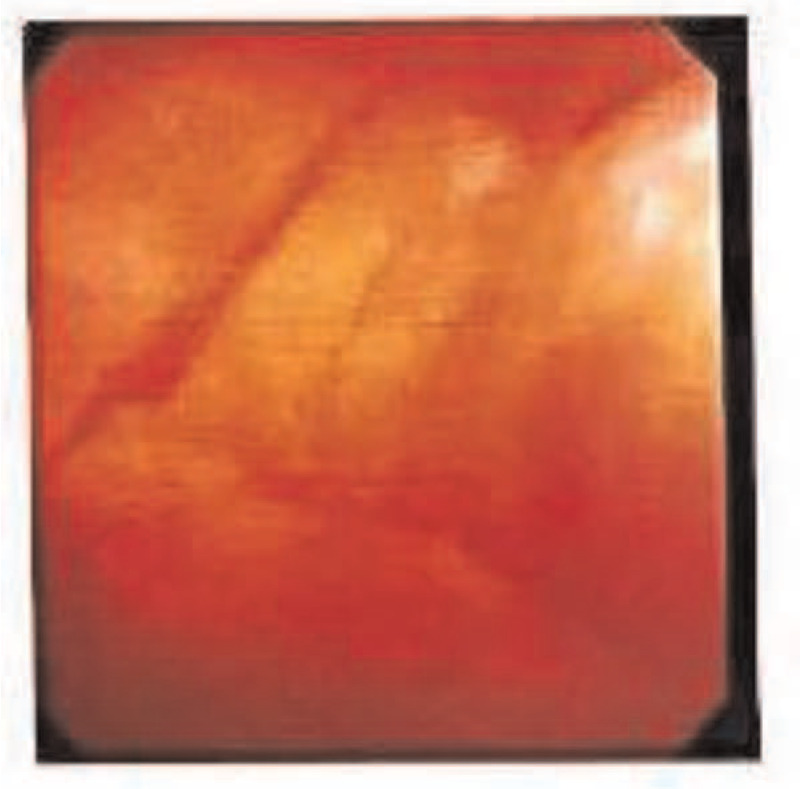
Findings by cystoscopy. The congestion of the posterior wall of the bladder showed obvious hyperemia.

In October 2018, the patient developed frequent urination, urgency, and dysuria with gross hematuria with blood clots and difficult urinating, especially at night and early in the morning. Upon routine urine examination, erythrocytes were in full field of view. There were no bacteria in urine. B-ultrasound showed no obvious abnormalities in both renal ureter and bladder. BKV-DNA in urine was 1.33 × 10^6^ copies/mL. Cystoscopy indicated obvious hyperemia and bleeding points in the mucosa of the posterior wall of the bladder (Fig. 1). Therefore, the patient was diagnosed as delayed HC of degree IV.

The treatment method included antiviral treatment of ganciclovir; large-dose fluid infusion. The daily infusion volume was calculated as 100 to 120 mL/kg/day, and it lasted for 24 hours; sodium bicarbonate was used to alkalize urine; diuresis: furosemide injection 20 mg/time, once every 6 hours (or adjusting the dosage according to the specific symptoms of the patient); bladder hemostasis with Prostaglandin E1. After treatment, the patient's symptoms of frequent urination, urgency, and dysuria did not improve but worsened. The patient had blood clots during morning and the symptoms of hematuria were obvious. The pain score was 4 points, and the ECOG score was also 4 points. Due to severe bleeding, recombinant human coagulation factor VIIa (2U) was used on January 3, 2019, with an interval of 8 hours. After 5 days of treatment, the patient's urinary red blood cell count remained repetitive and the symptoms of dysuria did not improve. The pain score was still 4 points. On January 17, 2019, cystoscopy showed that the congestion of the posterior wall of the bladder exhibited obvious hyperemia, indicating cystitis. Considering the poor results of conservative treatment, bilateral internal iliac artery embolization was performed on January 18, 2019. Hematuria symptoms improved after surgery, but the relief of dysuria was not obvious. After 5 days, the patient's urine red blood cells gradually increased again, and the interventional treatment for this patient was not effective. The patient was then treated with alkalization, fluid infusion and diuresis, and antiviral drugs. The symptoms of frequent urination, urgency, dysuria, and hematuria were not alleviated.

**Figure 2:**
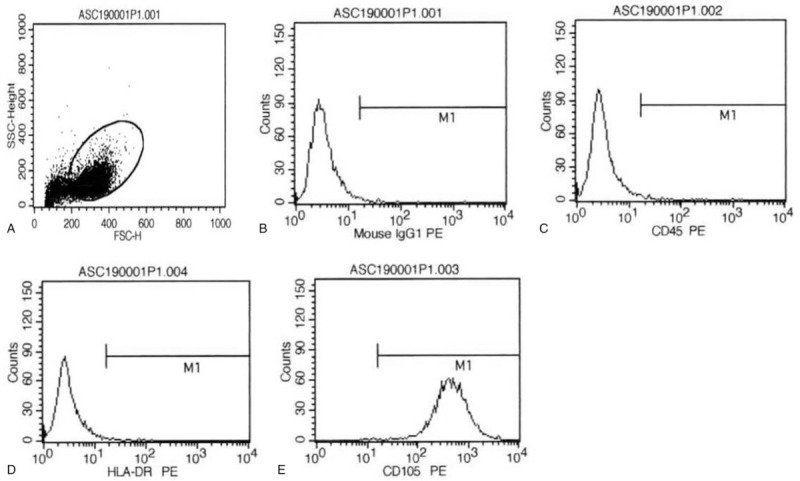
Flow cytometry measurement of MSCs. (A) Gating of MSCs. (B) Negative results with isotype antigen IgG1 PE. (C) Negative expression of CD45 on MSCs. (D) Negative expression of HLA-DR on MSCs. (E) Positive expression of CD105 on MSCs.

Due to severe HC and the long course of the disease, the life quality and mental state of the patient was seriously affected. The patient was then infused with ADSCs since February 22, 2019. Flow cytometry showed that CD105 was mainly expressed, and the transfusion was indeed MSCs (Fig. 2). The patient weighed 39 kg and the infusion volume was 3 units (60 mL), which equaled to 3 × 10^7^/kg. Urine samples were taken during the infusion process and 2, 4, 8, 12, and 24 hours after infusion. Urine routine tests were then performed daily. After the first infusion, the patient's urinary tract irritation symptoms and the gross hematuria improved, and the pain score was 3 points. Urinary red blood cells decreased from 21,491 cells/μL before infusion to 533 cells/μL. After 1 week, 3 units of ADSCs were infused again. Frequent urination, dysuria and urgency symptoms were further improved. The pain score reduced to 2 points. Urinary red blood cells decreased from 87/μL before infusion to 34/μL. One week later, the patient was infused with ADSCs for the third time, and the infusion volume climbed up to 4 units. The infusion process was smooth. The patient's urinary tract irritation symptoms were not obvious after the infusion. Pain score was 2 points. There was also a decrease in red blood cells, from 33/μL before infusion to 18/μL. The patient had no symptoms of urinary tract irritation, and no macroscopic or microscopic hematuria. These suggest that HC was resolved. The patient has been followed up for more than 2 years. She is currently alive and healthy and is regularly followed up in our hospital.

## 3 Discussion

HC is a common complication after HSCT. The mild symptoms are only microscopic hematuria, while the severe ones can cause urinary tract obstruction and even obstructive renal failure, which affects patients’ health and life quality. Severe GVHD and viral infection are the most important risk causes of delayed-onset HC.^[8]^ The possible reasons could be that GVHD can damage endothelial cells of parenchymal organs, causing damage to multiple organs (e.g., bladder). The recipient's bladder urothelial cells, especially the cells expressing viral antigens, are more likely attacked by donor lymphocytes. Another possible reason may be the application of immunosuppressive agents in the treatment of GVHD after allo-HSCT.^[4]^ In GVHD, the bladder mucosal epithelial cells may be destroyed, leading to the occurrence of HC. It is believed that HC is also related to virus infections, like BK,^[3,5]^ CMV, and influenza infections.^[9]^ The patient reported in this study had BK virus and JC virus infections, both of which showed high copy numbers.

MSCs can be easily isolated and enriched from a variety of tissues such as bone marrow, adipose tissue, or umbilical cord in vitro.^[10]^. The bone marrow is the gold standard tissue for MSC extraction. However, there are still some limitations for MSC extraction from bone marrow, such as the small number of bone marrow, and the decrease in the number of MSCs as the aging of the donor.^[11]^ On the contrary, the adipose tissue is abundant and easy to obtain, and can serve as a rich source of MSCs. In this study, the patient was infused with ADSCs, which showed significant curative effects.

In a previous study,^[12]^ MSCs were used to treat acute GVHD, and about half of patients were effectively treated. Acute GVHD is also an important pathogenic factor of HC.^[8]^ MSCs have low immunogenicity and exert immunomodulatory effects in vitro. They can inhibit the allogeneic activity of lymphocytes in culture of mixed lymphocytes,^[13]^ and are useful for tissue repair, cartilage defects, and tendons. MSCs have attracted great attention in the field of transplantation,^[14]^ which has the potential to directly differentiate into bladder mucosal epithelium and stimulate tissue repair through paracrine effects. Therefore, for patients with HC, the application of MSCs is expected to become a new treatment option.

To date, there is limited research on the use of MSCs in treating tissue toxicity caused by transplantation, such as severe delayed HC.^[12,15]^ In a previous study,^[6]^ MSC therapy was used in 7 severe HC cases of 33 HC patients and these 7 cases all received at least 1 MSC infusion. Ringden et al ^[12]^ showed that MSCs treatment could reduce the amount of blood transfusion and cure patients with mild HC. For severe HC patients, cystectomy was needed.^[16]^ Therefore, the early use of MSC to treat HC is of great importance.

However, the safety of MSCs treatment still needs attention. An open randomized controlled trial ^[17]^ reported that MSCs could prevent GVHD during HSCT, but the leukemia recurrence rate was significantly higher. Therefore, MSCs, as an emerging treatment, must be used very carefully before conducting large-scale clinical trials.

In conclusion, MSCs can be an effective treatment for severe HC, especially in patients with long duration and poor conventional treatment. It has good clinical application prospects. However, more clinical investigations are needed to further verify this.

## Author contributions

**Conceptualization:** Hailong Yuan.

**Data curation:** Ruixue Yang, Gang Chen.

**Formal analysis:** Ruixue Yang, Gang Chen, Maria Muhashi, Gulibadanmu Aizezi.

**Investigation:** Hailong Yuan.

**Methodology:** Ruixue Yang.

**Project administration:** Hailong Yuan.

**Software:** Ruixue Yang.

**Supervision:** Hailong Yuan.

**Writing – original draft:** Ruixue Yang, Gang Chen, Ming Jiang.

**Writing – review & editing:** Hailong Yuan.

## References

[1] Ringden O Remberger M Svahn BM. Allogeneic hematopoietic stem cell transplantation for inherited disorders: experience in a single center. Transplantation 2006;81:718–25. 16534474 10.1097/01.tp.0000181457.43146.36

[2] Lee GW Lee JH Choi SJ. Hemorrhagic cystitis following allogeneic hematopoietic cell transplantation. J Korean Med Sci 2003;18:191–5. 12692415 10.3346/jkms.2003.18.2.191 PMC3055015

[3] Korkmaz A Oter S Deveci S. Involvement of nitric oxide and hyperbaric oxygen in the pathogenesis of cyclophosphamide induced hemorrhagic cystitis in rats. J Urol 2003;170:2498–502. 14634459 10.1097/01.ju.0000085593.31396.d8

[4] Chong KT Hampson NB Corman JM. Early hyperbaric oxygen therapy improves outcome for radiation-induced hemorrhagic cystitis. Urology 2005;65:649–53. 15833500 10.1016/j.urology.2004.10.050

[5] García Ligero J Mora Peris B García García F. [Hemorrhagic cystitis caused by BK and JC polyomavirus in patients treated with bone marrow transplantation: clinical features and urologic management]. Actas Urol Esp 2002;26:104–10. 11989422 10.1016/s0210-4806(02)72741-1

[6] Wang Y Chen F Gu B. Mesenchymal stromal cells as an adjuvant treatment for severe late-onset hemorrhagic cystitis after allogeneic hematopoietic stem cell transplantation. Acta Haematol 2015;133:72–7. 25139500 10.1159/000362530

[7] Yuan H Jiang M Duan X. Related HLA-haploidentical T-cell replete peripheral stem cell transplantation with a reduced-intensity conditioning regimen to treat ANKL:a case report and literature review. Int J Clin Med 2019;12:6231–7.

[8] Ferrara JL Levy R Chao NJ. Pathophysiologic mechanisms of acute graft-vs.-host disease. Biol Blood Marrow Transplant 1999;5:347–56. 10595812 10.1016/s1083-8791(99)70011-x

[9] Ringdén O Horowitz MM Sondel P. Methotrexate, cyclosporine, or both to prevent graft-versus-host disease after HLA-identical sibling bone marrow transplants for early leukemia? Blood 1993;81:1094–101. 8427991

[10] Bernardo ME Locatelli F Fibbe WE. Mesenchymal stromal cells. Ann N Y Acad Sci 2009;1176:101–17. 19796238 10.1111/j.1749-6632.2009.04607.x

[11] Batsali AK Kastrinaki MC Papadaki HA. Mesenchymal stem cells derived from Wharton's Jelly of the umbilical cord: biological properties and emerging clinical applications. Curr Stem Cell Res Ther 2013;8:144–55. 23279098 10.2174/1574888x11308020005

[12] Ringden O Le Blanc K. Mesenchymal stem cells for treatment of acute and chronic graft-versus-host disease, tissue toxicity and hemorrhages. Best Pract Res Clin Haematol 2011;24:65–72. 21396594 10.1016/j.beha.2011.01.003

[13] Karlsson H Samarasinghe S Ball LM. Mesenchymal stem cells exert differential effects on alloantigen and virus-specific T-cell responses. Blood 2008;112:532–41. 18445691 10.1182/blood-2007-10-119370

[14] English K French A Wood KJ. Mesenchymal stromal cells: facilitators of successful transplantation? Cell Stem Cell 2010;7:431–42. 20887949 10.1016/j.stem.2010.09.009

[15] Ringdén O Uzunel M Sundberg B. Tissue repair using allogeneic mesenchymal stem cells for hemorrhagic cystitis, pneumomediastinum and perforated colon. Leukemia 2007;21:2271–6. 17611560 10.1038/sj.leu.2404833

[16] Giraud G Bogdanovic G Priftakis P. The incidence of hemorrhagic cystitis and BK-viruria in allogeneic hematopoietic stem cell recipients according to intensity of the conditioning regimen. Haematologica 2006;91:401–4. 16531266

[17] Ning H Yang F Jiang M. The correlation between cotransplantation of mesenchymal stem cells and higher recurrence rate in hematologic malignancy patients: outcome of a pilot clinical study. Leukemia 2008;22:593–9. 18185520 10.1038/sj.leu.2405090

